# Endoscopic Resection Before Surgery Does Not Affect the Recurrence Rate in Patients With High-Risk T1 Colorectal Cancer

**DOI:** 10.14309/ctg.0000000000000336

**Published:** 2021-04-12

**Authors:** Hiroyuki Takamaru, Yutaka Saito, Masau Sekiguchi, Masayoshi Yamada, Taku Sakamoto, Takahisa Matsuda, Shigeki Sekine, Hiroki Ochiai, Shunsuke Tsukamoto, Dai Shida, Yukihide Kanemitsu

**Affiliations:** 1 Endoscopy Division, National Cancer Center Hospital, Tokyo, Japan;; 2 Cancer Screening Center, National Cancer Center, Tokyo, Japan;; 3 Pathology Division, National Cancer Center Hospital, Tokyo, Japan;; 4 Department of Colorectal Surgery, National Cancer Center Hospital, Tokyo, Japan.

## Abstract

Patients with high-risk T1 colorectal cancer (CRC) after endoscopic resection (ER) should undergo surgery in view of the risk of lymph node metastasis. Although additional surgery can potentially prevent recurrence, there is a paucity of data and longitudinal studies exploring this potential. Hence, this study aimed to evaluate the prolonged influence of ER before additional surgery on recurrence in T1 CRC.

**METHODS:**

Between January 2004 and October 2015, 162 patients who underwent secondary surgery (SS) after ER ([ER + SS] group) and 392 consecutive patients with T1 CRC who underwent primary surgery at our institution were retrospectively analyzed. Recurrence was analyzed in these 2 groups. High-risk CRC patients were histologically defined according to the Japanese Society for Cancer of the Colon and Rectum guidelines (2016) for the treatment of CRC. Data were analyzed based on clinical and histological features, including lymph node metastasis, and the number of lymph nodes evaluated.

**RESULTS:**

The recurrence rate was comparable between the ER + SS and primary surgery groups, with no significant difference (*P* = 0.625, log-rank test). There was no significant difference in the recurrence in patients receiving adjuvant chemotherapy in both groups (7.4% vs 10.4%, *P* = 0.27). The difference in the mean number of lymph nodes dissected between both groups was also not significant (24.3 vs 25.3, *P* = 0.43).

**DISCUSSION:**

There was no significant difference in recurrence rates between patients undergoing ER before surgery and those undergoing primary surgery for high-risk T1 CRC. Hence, ER may be acceptable for high-risk T1 CRC.

## INTRODUCTION

T1 colorectal cancer (CRC) with a low risk of lymph node metastasis (LNM) can be treated by endoscopic resection (ER) without additional surgery (1–3). Patients with T1 CRC that have unfavorable histological features such as depth >1,000 μm or positive lymphatic invasion are recommended additional treatment by secondary surgery (SS) to avoid LNM (4–7). However, these histological factors become apparent only after ER is performed. Some studies suggest that inadequate endoscopic treatment for T1 CRC accelerates the malignant potential of CRC and imputes a high risk of metastatic disease (8,9). By contrast, other studies showed no adverse effect of endoscopic treatment on long-term outcomes (1–3,10–13). We rarely observe recurrences after ER followed by SS for T1 CRC in clinical practice. Therefore, ER before SS may be acceptable. However, previous studies have reported several limitations, including a low *en bloc* resection ratio (1,14), lack of data on adjuvant chemotherapy for patients with LNM (10,12,15–17), or evaluation of only a small number of lymph nodes postsurgical resection (13,18). In this context, the effect of ER performed before SS on long-term recurrence remains unclear. Therefore, this study aimed to analyze whether ER before SS affects long-term recurrence in patients with high-risk T1 CRC while evaluating the number of resected lymph nodes and adjuvant chemotherapy.

## METHODS

### Selection and description of participants

**Figure 1.**
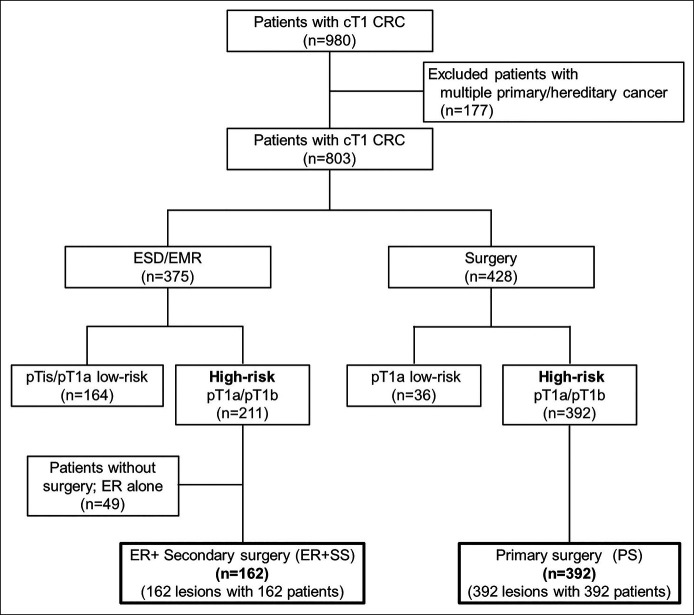
Flow chart of the patient selection process. A total of 162 patients treated with ER followed by additional surgery (secondary surgery group) and 392 patients treated with surgery alone (PS group) were analyzed. cT1, clinical T1; CRC, colorectal cancer; ESD, endoscopic submucosal dissection; EMR, endoscopic mucosal resection; pTis, pathological intramucosal neoplasia; pT1a, pathological submucosal invasion <1,000 μm; pT1b, pathological submucosal invasion ≥1,000 μm; ER, endoscopic resection.

We included patients with clinical T1 CRC who were treated using ER, ER followed by SS, or primary surgery (PS) between January 2004 and October 2015. Patients with high-risk T1 CRC who were treated by SS after ER (ER + SS) and PS were retrospectively analyzed. Figure 1 shows the flowchart of the patient selection criteria used in this study. During the study period, 980 patients with clinical T1 CRC were treated at the National Cancer Center Hospital, Tokyo, Japan. Patients with a history of previous CRC, inflammatory bowel disease, family history of hereditary or familial CRC (diagnosed as familial adenomatous polyposis or Lynch syndrome), previous colectomy, or those who were previously treated for another malignancy before surgery for CRC were excluded, as we intended to analyze only the risk of distant metastasis in CRC. Based on these criteria, 177 patients were excluded. Of the remaining 803 patients, 375 were treated with ER, and 428 patients with clinical T1 CRC were treated with surgery. After excluding histological Tis (intramucosal neoplasia/tumor *in situ*) or low-risk T1a (<1,000 μm without unfavorable histological factors) from each group and 49 patients who were not treated with SS after ER, 162 patients treated by ER followed by SS (ER + SS) and 392 patients treated with PS were analyzed.

### Ethical statement

All procedures in this study involving human participants were performed according to the ethical standards of the Institutional Review Board of the National Cancer Center Hospital (IRB number: 2016-447) and with the 1964 Helsinki Declaration and its later amendments or comparable ethical standards. The requirement for informed consent was waived by the ethics committee owing to the retrospective nature of this study. The National Cancer Center Hospital opted out from the opportunity to reject this study.

### Indications for endoscopic mucosal resection/endoscopic submucosal dissection

We defined the indications for endoscopic mucosal resection (EMR) or endoscopic submucosal dissection (ESD) according to the JGES guidelines for colorectal ESD/EMR (19). Briefly, colorectal carcinomas with suspected depth of Tis or T1 were treated by ER. Lesions <20 mm in size without a nonlifting sign were an indication for EMR, and ESD was performed when lesions were >20 mm. Lesions <20 mm in size with the nonlifting sign were also eligible for ESD. We used magnifying endoscopy with crystal violet staining to ascertain the depth of invasion before the procedure. The depth of invasion was defined as Tis or T1a without an invasive pit pattern (20).

### Procedures

The ESD technique is well described in literature (21). Briefly, colorectal ESD is usually performed using a ball-tip bipolar needle knife with a water jet function (Jet B-knife; XEMEX, Tokyo, Japan) or an insulation-tipped electrosurgical knife (IT knife nano, KD-612Q; Olympus Medical Systems, Tokyo, Japan). After submucosal injection, we dissected the submucosal layer using knives. The aforementioned methods ensure a high *en bloc* resection rate needed for the treatment of T1 CRC by ER.

We used the SnareMaster snare (10, 20, or 25 mm, Olympus Medical Systems) or double-loop snare (Dualoop, Medico's Hirata, Tokyo, Japan) for EMR. The choice of the snare was decided according to the size of the lesion and the endoscopist's preference.

### Indication of SS

Once ER was completed, the requirement of SS was evaluated according to the histological diagnosis as per the Japanese classification in each period (22–24). High-risk CRC was defined when one of the following factors was observed during the histological examination of the lesions: (i) positive for vertical margin; (ii) depth of T1b (>1,000 μm); (iii) T1a (<1,000 μm) with positive lymphatic or venous invasion; (iv) T1a with poorly differentiated, mucinous, or signet ring cell component; or (v) T1a with budding grade 2/3. Criterion “a” was considered as an absolute indication for additional surgery with lymph node dissection, while the other criteria (b, c, d, and e) were considered as relative indications for SS (7). The final treatment strategy was decided after a multidisciplinary meeting with a surgeon and another consultation of the patient during which the outcomes of the meeting were discussed.

### Data evaluation

We evaluated the age, sex, size of the lesion, macroscopic features, location of the lesion, endoscopic procedure (EMR or ESD, *en bloc* resection ratio, and the ratio of positive vertical margin), histological findings (predominant histology, depth of invasion, lymphatic invasion, venous invasion, and LNM), observation period, period from the initial diagnosis to surgery, and recurrence rate, including local and distant recurrence. Tumor size was histologically defined as the largest diameter of the lesions.

Medical records of each patient were reviewed for clinical and histological findings. The macroscopic features were endoscopically determined and divided into 2 groups: 1) protruded type [type 0-Ip/0-Isp/0-Is or a combination of these 2 types (0-Is + IIa)] and 2) flat or depressed type [0-IIa, 0-IIc, or a combination of these 2 types (0-IIa + IIc)]. Lesions that were a mixture of protruded and depressed types were considered depressed type [0-Is + IIc].

Histopathological examination was performed following the recommendations of the Japanese classification of colorectal carcinoma and Japanese Society for Cancer of the Colon and Rectum guidelines for treating CRC in each era (4,22–26). The depth of invasion was also graded according to the same Japanese classification of colorectal carcinoma (22–24). The method used to measure the distance of submucosal invasion is as follows: When histological findings revealed complete invasion of the muscularis mucosa by the tumor, the distance of submucosal invasion was measured from the top of the lesion to the deepest part of the lesion; conversely, when the muscularis mucosa was histologically retained, the distance was measured from the level of muscularis mucosa to the deepest part of the lesion. For histological evaluations, resected specimens during surgery were sectioned at 5-mm intervals, whereas endoscopically resected specimens were sectioned at 2-mm intervals. When the diagnosis of lymphatic involvement was inconclusive using hematoxylin-eosin (H&E)-stained sections, immunohistochemistry was performed with the monoclonal antibody D2-40 and/or Victoria blue or Elastica van Gieson staining.

### Follow-up of the patients and definitions of recurrence

After PS or SS, we checked serum carcinoembryonic antigen levels every 3 months, while total computed tomography (CT) of the chest, abdomen, and pelvis was examined every 6 months. Total colonoscopy (TCS) was scheduled annually for the first 3 years. Thereafter, CT examination and TCS were considered annually.

The recurrence of CRC was defined as CRC that was histologically confirmed by a biopsy at or near the original tumor location, or distant metastases identified by CT or other imaging techniques, including magnetic resonance imaging or abdominal ultrasound.

### Statistical analysis

Results are expressed as mean ± SD or median and range. Continuous variables were compared using the Student *t* test. Categorical variables were compared between both groups using the χ^2^ test or Fisher exact test, as appropriate. To evaluate long-term recurrence, we used the Kaplan–Meier method to estimate the recurrence-free curve and Cox proportional hazards models to estimate the hazard ratios and 95% confidence intervals. One-to-one propensity score matching was used to compensate for selection bias and potential confounding between the ER + SS and PS groups (27). We assessed histological factors that were previously reported to have influenced recurrence (4,28,29). To calculate the propensity score, we used confounders that differ between the 2 groups and are believed to be clinically associated with recurrence according to the previous report (1), to adjust for the background. Nearest neighbor matching was performed to create a matched sample with a caliper width of 0.02.

A two-tailed *P* value < 0.05 was considered statistically significant. Statistical analysis was performed using JMP SAS version 14.3.0 (SAS Institute, Cary, NC) and R software version 3.4.3 (The R Foundation Vienna, Austria).

## RESULTS

### There was no difference in the overall recurrence analysis

**Figure 2.**
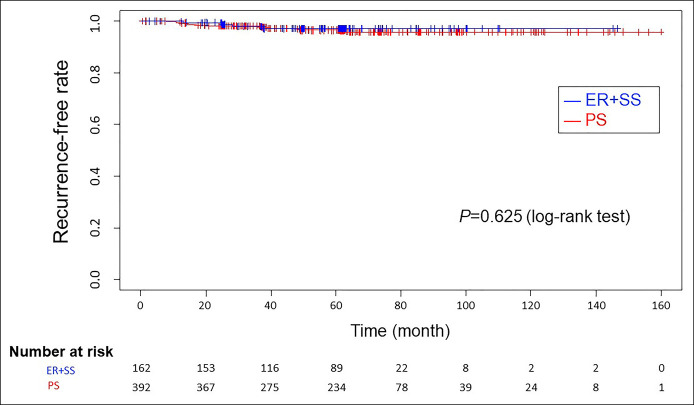
Recurrence-free rate of patients in the 2 groups. The 2 groups showed no differences in recurrence (log-rank test, *P* = 0.625). The median observation period was 59.5 months (range: 5.2–151.0) in the secondary surgery group (ER + SS) and 63.3 months (range: 0.6–166.2) in the PS group. ER, endoscopic resection; SS, secondary surgery; PS, primary surgery.

There was no significant difference in recurrence between the ER + SS and PS groups (*P* = 0.625, log-rank test) (Figure 2). During a median follow-up duration of 59.1 months, recurrence developed in 17/559 patients. Of these 17 patients, 4 (2.5%) were in the SS group and 13 (3.3%) were in the PS group (Table 1). Recurrence was detected at 12–60 months after surgery. All 4 patients in the ER + SS group had distant metastases, whereas there were 10 distant metastases, 2 regional lymph node metastases, and 1 local recurrence in the 13 patients in the PS group. The details of all patients with recurrence are summarized in Table, Supplementary Digital Content 1, http://links.lww.com/CTG/A560.

**Table 1.** Recurrence of ER before secondary and primary surgery

Recurrence	ER + SS (n = 162)	Surgery (n = 392)	*P* Value
(+) [95% C.I.]	4 (2.5%) [0.1%–6.2%]	13 (3.3%) [1.9%–5.6%]	0.79^a^
(−)	158 (98.1%)	379 (96.7%)	

Both the groups showed no significant differences in the number of recurrences (*P* = 0.79).

a The Fisher exact test.

CI, confidence interval; ER, endoscopic resection; SS, secondary surgery.

### Patient demographics

In the SS group, the lesions were larger (27.5 ± 20.0 vs 22.3 ± 15.2 mm, *P* < 0.01) and had more protruded components than those in the PS group, including 0-Is (85 patients, 52.5% vs 134 patients, 34.2%; *P* < 0.01), and more patients were diagnosed with T1a (27 patients, 16.7% vs 12 patients, 3.1%; *P* < 0.01). The mean period from initial diagnosis to surgery was longer in the ER + SS group than in the PS group (2.4 vs 0.8 months, *P* < 0.01) (Table 2).

**Table 2.** Demographic data of the patients and lesions

	ER + SS (n = 162)	Surgery (n = 392)	*P* Value
Sex			
Male	95	221	0.62^b^
Female	67	171	
Age, yr (mean ± SD)	62.3 ± 10.5	63.1 ± 11.0	0.41^d^
Lesion size			
Endoscopically estimated	29.1 ± 20.7	24.0 ± 12.9	<0.01^d^
Histologically evaluated	27.5 ± 20.0	22.3 ± 15.2	<0.01^d^
Macroscopic feature			<0.01^b^
Ip/Isp/Is/Is + IIa	85 (52.5%)	134 (34.2%)	
IIa/IIc/IIa + IIc/Is + IIc	77 (47.5%)	258 (65.8%)	
Location of the lesion			0.47^b^
Proximal colon (C-T)	56 (34.6%)	123 (31.4%)	
Distal colon (D-Rs)	60 (37.0%)	137 (34.9%)	
Rectum (Ra-Rb)	46 (28.4%)	132 (33.7%)	
Mean period to surgery [mo]	2.4 ± 1.5	0.8 ± 1.0	<0.01^d^
Lymphatic invasion			<0.01^b^
(+)	51 (30.9%)	77 (19.6%)	
(−)	111 (69.1%)	31 (80.4%)	
Venous invasion			0.02^b^
(+)	35 (21.6%)	125 (31.9%)	
(−)	127 (78.4%)	267 (68.1%)	
Predominant histology			<0.01^b^
Well-differentiated tubular adenocarcinoma	149 (92.0%)	300 (76.5%)	
Moderately differentiated tubular adenocarcinoma	12 (7.4%)	83 (21.1%)	
Poorly differentiated tubular adenocarcinoma	0	3 (0.8%)	
Mucinous	1 (0.6%)	2 (0.5%)	
Papillary adenocarcinoma	0	4 (1.0%)	
Risk factor of LNM^a^			0.31^b^
(+)	20 (12.4%)	61 (15.7%)	
(−)/unknown	142 (87.6%)	327 (84.3%)	
Depth of invasion			<0.01^b^
pT1a	27 (16.7%)	12 (3.1%)	
pT1b	135 (83.3%)	380 (96.9%)	
Recurrence			0.79^c^
(+)	4 (2.5%)	13 (3.3%)	
(−)	158 (97.5%)	379 (96.7%)	

A higher number of patients were diagnosed with T1a, and most lesions were histologically diagnosed as well-differentiated adenocarcinoma in the secondary surgery group.

ER, endoscopic resection; LNM, lymph node metastasis; SS, secondary surgery.

a Risk factors of LNM included histological findings of the poorly differentiated component, mucinous adenocarcinoma component, signet ring cell component, budding grade 2 or 3, pT1a: pathologically evaluated submucosal invasion <1,000 μm, pT1b: pathologically evaluated submucosal invasion ≥1,000 μm.

b The chi-square test.

c The Fisher exact test.

d The Student *t* test.

### A number of lymph nodes were resected and patients in both groups received adequate adjuvant therapy

The difference in the mean number of dissected lymph nodes between both groups was also insignificant (24.3 vs 25.3, *P* = 0.43, Table 3). Patients with LNM received adjuvant chemotherapy with oral 5-fluorouracil (FU) for 6 months. There was no significant difference in the number of patients with and without adjuvant therapy between both groups (12 patients in SS, 7.4% vs 41 patients in PS, 10.4%; *P* = 0.27; Table 3). Of the 12 patients who received adjuvant chemotherapy in the SS group, 2 were graded as pStage I; however, because of the high-grade atypia in their histological features, adjuvant therapy was considered. One patient with pStage III refused adjuvant chemotherapy. Furthermore, there was no significant difference in the LNM prevalence at the time of surgery between both groups (10 patients, 6.2% vs 42 patients, 10.7%; *P* = 0.11). In the SS group, the frequency of lymphatic invasion was higher (51, 30.9% vs 77, 19.6%; *P* < 0.01), while that of venous invasion (35, 21.6% vs 125, 31.9%; *P* = 0.02) was lower than in the PS group (Table 2).

**Table 3.** Histological staging and adjuvant treatment

	ER + SS (n = 162)	Surgery (n = 392)	*P* Value
Number of dissected LNs (mean ± SD)	24.3 ± 13.8	25.3 ± 12.8	0.43^c^
LNM			0.11^d^
(+)	10 (6.2%)	42 (10.7%)	
(−)	152 (93.8%)	350 (89.3%)	
pStage			0.22^d^
Stage I	152 (93.8%)	350 (89.3%)	
Stage IIIa	7 (4.3%)	36 (9.2%)	
Stage IIIb	3 (1.9%)	5 (1.3%)	
Stage IV^a^	0	1 (0.3%)	
Adjuvant treatment			0.27^d^
Yes	12^b^ (7.4%)	41 (10.4%)	
No	150 (92.6%)	351 (89.6%)	

There was no significant difference in the ratio of patients with and without adjuvant therapy between the 2 groups.

LN, lymph node; LNM, lymph node metastasis.

a Stage IV due to para-aortic LNM.

b Two patients treated by adjuvant chemotherapy even in pStage I because of a histological unfavorable evaluation.

c The student *t* test.

d The chi-square test, pStage: pathological staging.

### Cox regression analysis revealed risk factors for recurrence of T1 CRC

Multivariate analysis for predicting long-term recurrence using the Cox hazard model showed that positive venous invasion and location in the rectum were independent risk factors for recurrence of high-risk T1 CRC (Table 4). We performed a stratified analysis of the location and found that the recurrence rate was significantly higher in the rectum in both ER + SS and PS groups (log-rank test; *P* = 0.032, *P* < 0.01, respectively). The 5-year recurrence rate was higher in the rectum than the colon (8.0% [95% CI 2.6–22.2] vs1.0% [0.1–6.5], respectively) in the ER + SS group. The recurrence rate in the rectum was also higher in the PS group (8.4% [95% CI 4.4–15.5] vs 1.2% [0.4–3.6], respectively). The Kaplan–Meier curves for each group are shown in a supplementary table, http://links.lww.com/CTG/A560 (Figure, Supplementary Digital Content 2, http://links.lww.com/CTG/A561).

**Table 4.** Multivariate analysis

	Hazard ratio	95% C.I.	*P* Value
Location of the lesion			<0.01
Colon	ref	[2.1–20.6]	
Rectum	6.56		
Vessel invasion			<0.01
(−)	ref		
(+)	4.13	[1.5–11.6]	
Treatment			0.950
ER + SS	ref		
Surgery	0.95	[0.3–3.1]	

The Cox hazards model analysis revealed that positive vessel invasion and location in the rectum were independent risk factors for the recurrence of high-risk T1 CRC.

CI, confidence interval; ER, endoscopic resection; SS, secondary surgery.

### A high ratio of *en bloc* resection was presented by ER

Ninety patients (55.3%) were treated using the ESD technique, and others were treated using EMR. The *en bloc* resection ratio for all ERs was 92.0%. Of the 149 patients with *en bloc* resection, 32 (19.8%) showed a positive vertical margin (VM), although no recurrence was observed in these patients (Table 5). Three patients with intraoperative perforation had no recurrence, and the mean of the 3 survival periods was 77 months (data not shown).

**Table 5.** Short-term outcome of endoscopic resection

	ER + SS (n = 162)
Procedure	
ESD	90 (55.3%)
EMR/polypectomy	72 (44.7%)
Resection	
*En bloc*	149 (92.0%)
Piecemeal	13 (8.0%)
Perforation	
Intraoperative	3 (1.9%)
Delayed	0
Resected margin	
VM (−)	130 (80.2%)
VM (+)	32 (19.8%)

Of all ER patients, 55.3% were treated using the ESD technique.

ER, endoscopic resection; ESD, endoscopic submucosal dissection; EMR, endoscopic mucosal resection; SS, secondary surgery; VM, vertical margin.

### The propensity score–matched study showed the same tendency of recurrence between both groups

A propensity score was acquired for each lesion using the potential confounders described in the "Methods" section. The one-to-one nearest neighbor match indicated 143 matching pairs. Only the time to surgery was different between both groups. All other characteristics of the lesions and patients, including the number of assessed lymph nodes postoperatively, were comparable between both groups. Moreover, the difference in the LNM prevalence in the PS and SS groups was not significant. Therefore, pathological staging and subsequent adjuvant therapy were also adjusted by this matching. The long-term outcomes (recurrence rate in the total follow-up period and Kaplan–Meier analysis of recurrence or metastasis) were similar between both groups (Table, Supplementary Digital Content 3, http://links.lww.com/CTG/A562; and Figure, Supplementary Digital Content 4, http://links.lww.com/CTG/A563).

## DISCUSSION

In this study, there was no difference in the recurrence rates between patients undergoing ER + SS than those undergoing PS alone for high-risk T1 CRC. The results of this study may help ascertain the long-term outcomes in patients with high-risk T1 CRC. This study addresses the question whether ER may affect the recurrence of high-risk T1 CRC, which should be treated surgically. Many factors are known to contribute to long-term outcomes, including recurrence after T1 CRC treatment. First, stage III CRC needs to be treated with adjuvant chemotherapy because this may significantly affect recurrence (18,30,31). In this study, all the patients were investigated for treatment with adjuvant chemotherapy, and there was no significant difference between both groups. Second, it is important to consider the number of dissected lymph nodes to determine the histological stage (4). In this study, we evaluated approximately 24 lymph nodes, which enabled us to evaluate LNM precisely. Even with such a meticulous examination of the lymph nodes, the LNM prevalence was 6.2% and 10.7% in the SS and PS groups, respectively, which is consistent with those in previous reports (1–3,12,13). However, in our study, the recurrence rates after SS and PS was 2.5% and 3.3%, respectively; this rate is significantly lower than that in previous studies with recurrence rates of 4.4% and 7.2%, respectively (1). Third, LNM could be underestimated when fewer lymph nodes are dissected. Stage III CRC can be misdiagnosed as stage I when the number of resected and assessed lymph nodes is insufficient. Thus, such a patient may be denied treatment with adjuvant chemotherapy, subsequently leading to a high recurrence rate.

We followed up patients for a median of 59.1 months, and many patients showed recurrence between 12 and 60 months. This follow-up period was sufficient to evaluate the long-term recurrence after surgery in patients with high-risk T1 CRC. When patients were first treated with surgery, treatment was immediately performed after diagnosis. However, in the ER + SS group, patients underwent surgery after a histological examination from the pathologists, which reported an unfavorable histological feature in the resected specimens. The follow-up period in the ER + SS group was significantly longer than in the PS group. However, there were no significant differences in the long-term recurrence rates between both groups. Next, we performed a subanalysis of the ER + SS group with and without recurrence to investigate the relationship between the period from endoscopic treatment to additional surgical resection and the recurrence rate. There was no difference in the mean period from ER to additional surgery between the recurrence-free and recurrence groups (2.2 months [range 0.2–9.6] vs1.8 months [range 1–2.6], *P* = 0.43). It is generally speculated that the longer the time to additional surgery, the higher the probability of recurrence. Most patients underwent additional resection within 6 months in our study; however, the effect of the time to surgery was small.

In addition, there was no significant difference in overall survival or recurrence-free survival between the ER + SS and PS groups (Figure, Supplementary Digital Content 5, http://links.lww.com/CTG/A564). These factors indicate that a longer time to surgery has no effect on long-term recurrence or survival after an ER.

We found that recurrence was significantly higher in the rectum irrespective of the treatment choice (Figure, Supplementary Digital Content 2, http://links.lww.com/CTG/A561). This supports previous reports that the location of the rectum itself is a risk factor for recurrence. Recurrence should be anticipated, regardless of the treatment strategy. In addition, the Kaplan–Meier curve showed that recurrence occurred within 5 years, suggesting that a 5-year follow-up is necessary.

Compared with the ER alone group, the ER + SS group had better survival. Of the 49 patients treated with ER alone, only 2 had recurrence; both patients were in their 40s and had recurrence in the rectum. The time of recurrence was 3–6 years after endoscopic treatment (Figure, Supplementary Digital Content 6, http://links.lww.com/CTG/A565). By contrast, recurrences were mostly found in the rectum, in patients aged 39–75 years, and mostly occurred 1–3 years after surgery in the SS group (Table, Supplementary Digital Content 1, http://links.lww.com/CTG/A560). However, statistical significance was not achieved because of the small number of patients treated with ER alone. Some patients who were followed up with ER alone developed recurrence after a relatively long period; however, it is difficult to say this with 100% certainty because of the small sample size. In such patients, follow-up beyond 5 years should be considered. Future studies, including multicenter studies, are needed to evaluate other outcomes.

One of the strengths of our study was that the data were based on a high ratio of *en bloc* resection, which was 92.0%, when both EMR and ESD techniques that enabled a precise evaluation were used. The *en bloc* resection by endoscopy enables a precise histological evaluation of ER + SS. In addition, a careful lymph node evaluation provides accurate staging after surgery (30–34).

This study had several limitations. First, it was a single-center retrospective analysis; therefore, it had a potential for bias. However, as a high-volume center, standards were used for both procedural techniques of endoscopic treatment and surgery, and histological evaluation. Second, differences in background characteristics between both groups may have caused the selection bias. This is partially because preoperative depth diagnosis between pathologically diagnosed T1a (pT1a) and pT1b is still challenging, despite the use of magnified endoscopic techniques. In clinical practice, most clear pT1b CRC cases are diagnosed by conventional endoscopy and treated with surgery, whereas endoscopic treatment was performed in clinically diagnosed T1 CRC, which is difficult to diagnose preoperatively. Only few pT1b CRCs may be treated by ER because of preoperative diagnosis. Thus, an accurate diagnosis of endoscopic depth before endoscopic treatment is critical. Third, the surgical treatment was decided based on the preoperative diagnosis. In addition, patients who refused or failed to undergo surgery for various reasons were excluded. Therefore, there was a selection bias in the study. We performed a propensity matched analysis, and almost all baseline characteristics were adjusted (Table, Supplementary Digital Content 3, http://links.lww.com/CTG/A562). After matching, we consider our results reliable to an extent since the long-term outcomes by Kaplan–Meier analysis were similar to those before matching.

In conclusion, the study found no difference in the rates of local, lymph node, or distant metastases between patients undergoing surgery post-ER and those undergoing surgery alone. Moreover, ER may be acceptable in patients with high-risk T1 CRC before surgery.

## CONFLICTS OF INTEREST

**Guarantor of the article:** Yutaka Saito, MD, PhD.

**Specific author contributions:** H.T., Y.S., and Y.K.: study concept and design. All authors: acquisition of data and critical revision of the manuscript for important intellectual content. H.T. and Y.S.: analysis and interpretation of data. H.T.: statistical analysis and drafting of the manuscript. Y.S.: obtained funding. Y.S. and Y.K.: study supervision. All authors have read and approved the final draft of the manuscript.

**Financial support:** This work was supported in part by the National Cancer Center Research and Development Fund (25-A-12, 28-K-1, and 29-A-13), obtained by Y.S.

**Potential competing interests:** None reported.

**Study Highlights**

**WHAT IS KNOWN**

✓ Additional surgery after endoscopic resection (ER) for T1 colorectal cancer reduces the risk of lymph node metastasis.
✓ Large-scale data on recurrence of ER followed by secondary surgery are unavailable.

**WHAT IS NEW HERE**

✓ Compared with primary surgery, ER after secondary surgery showed no effect on recurrence rates.
✓ Approximately 24–25 lymph nodes were dissected in both the primary and secondary surgery groups.
✓ *En bloc* ER was performed in 92.0% of the cases.

**TRANSLATIONAL IMPACT**

✓ ER before secondary surgery may be acceptable for high-risk T1 colorectal cancer.

## Supplementary Material

SUPPLEMENTARY MATERIAL
