# Supplementary material for: Endoscopic Resection Before Surgery Does Not Affect the Recurrence Rate in Patients With High-Risk T1 Colorectal Cancer
Source: Clin Transl Gastroenterol. 2021 Apr 12;12(4):e00336. doi: 10.14309/ctg.0000000000000336 (PMC8043730; doi:10.14309/ctg.0000000000000336)
Supplement: SUPPLEMENTARY MATERIAL [file ct9-12-e00336-s001.docx]

**Table, Supplementary Digital Content 1. Characteristics of the 17 patients with recurrence**

| **Patient** | **Age** | **Sex** | **Location** | **Interval to recurrence*** | **CRD** | **Primary treatment** | **Histology** | **Ly** | **V** | **VM** | **Other risk factors of LNM**** | **En bloc (ER)** | **Metastatic feature** |
| --- | --- | --- | --- | --- | --- | --- | --- | --- | --- | --- | --- | --- | --- |
| 1 | 48 | M | C | 24.8 | No | ESD | Well | (-) | (+) | (-) | (-) | En bloc | Liver |
| 2 | 58 | M | D | 12.3 | No | Surgery | Well | (-) | (-) | n/a | Unknown | n/a | Anastomosis |
| 3 | 56 | F | S | 10.8 | Yes | Surgery | Well | (+) | (+) | n/a | (-) | n/a | Peritoneum |
| 4 | 71 | M | S | 13.7 | No | Surgery | Mod | (-) | (-) | n/a | (-) | n/a | Liver |
| 5 | 59 | M | Rb | 63.0 | Yes | Surgery | Mod | (-) | (-) | n/a | Unknown | n/a | Lung |
| 6 | 72 | M | Rb | 37.0 | Yes | Surgery | Well | (-) | (+) | n/a | Unknown | n/a | Lung |
| 7 | 60 | M | Rb | 20.2 | Yes | Surgery | Well | (+) | (+) | n/a | Unknown | n/a | Lung |
| 8 | 67 | M | Rb | 7.8 | No | Surgery | Mod | (-) | (+) | n/a | (-) | n/a | Liver |
| 9 | 72 | M | Rb | 47.7 | No | Surgery | Mod | (+) | (+) | n/a | Unknown | n/a | Lung |
| 10 | 61 | F | Rb | 48.8 | No | Surgery | Well | (-) | (-) | n/a | (-) | n/a | L/N |
| 11 | 39 | M | Rb | 37.2 | No | ESD | Well | (-) | (-) | (-) | (+) | En bloc | Lung |
| 12 | 62 | M | Ra | 28.2 | Yes | ESD | Well | (-) | (+) | (-) | Unknown | En bloc | Para Ao L/N |
| 13 | 39 | F | Rb | 14.3 | No | Surgery | Well | (-) | (+) | n/a | (-) | n/a | Liver |
| 14 | 75 | M | Ra | 12.9 | Yes | pEMR | Well | (-) | (-) | (-) | (+) | Piecemeal | Liver |
| 15 | 65 | M | Rb | 38.9 | No | Surgery | Well | (+) | (+) | n/a | Unknown | n/a | Lung |
| 16 | 53 | M | Ra | 17.0 | No | Surgery | Well | (-) | (+) | n/a | (-) | n/a | Liver |
| 17 | 64 | F | Rb | 36.4 | No | Surgery | Well | (+) | (+) | n/a | Unknown | n/a | L/N |

Of the total 17 lesions, 13 were located in the rectum, 11 invaded the vessels, and 12 had distant metastases (liver or lungs).

CRD, cancer-related death; LNM, lymph node metastasis; ** Risk factors for LNM included histological findings of the poorly differentiated component, mucinous adenocarcinoma component, signet ring cell component, or budding grade 2 or 3; Ly, lymphatic invasion; V, venous invasion; VM, vertical margin; ER, endoscopic resection; C, cecum; D, descending colon; S, sigmoid colon; Ra, upper rectum; Rb, lower rectum. ESD; endoscopic submucosal dissection, pEMR; piecemeal EMR, well; tubular adenocarcinoma well differentiated, mod; tubular adenoma moderately differentiated, Ao; aorta, L/N; lymph node, n/a; not applicable (because of primary surgery)
