# Supplementary material for: Endoscopic Resection Before Surgery Does Not Affect the Recurrence Rate in Patients With High-Risk T1 Colorectal Cancer
Source: Clin Transl Gastroenterol. 2021 Apr 12;12(4):e00336. doi: 10.14309/ctg.0000000000000336 (PMC8043730; doi:10.14309/ctg.0000000000000336)
Supplement: SUPPLEMENTARY MATERIAL [file ct9-12-e00336-s002.docx]

**Table, Supplementary Digital Content 3. Propensity score-matched analysis**

|  | ER+SS | Surgery | *P* value |
| --- | --- | --- | --- |
|  | (n = 143) | (n = 143) |  |
| Gender |  |  |  |
| Male | 81 (56.6%) | 75 (52.5%) | 0.48^†^ |
| Female | 62 (43.4%) | 68 (47.6%) |  |
| Age, years (mean ± SD) | 62.3 ± 10.8 | 62.9 ± 11.3 | 0.65^‡^ |
| **Histological size of the lesion**** | 27.2 ± 18.5 | 26.8 ± 19.2 | 0.87^‡^ |
| **Macroscopic feature**** |  |  | 0.41^†^ |
| Ip/Isp/Is/Is+IIa | 73 (51.1%) | 80 (55.9%) |  |
| IIa/IIc/IIa+IIc/Is+IIc | 70 (49.0%) | 63 (44.1%) |  |
| Location of the lesion |  |  | 0.42^†^ |
| Proximal colon (C-T) | 53 (37.6%) | 44 (30.8%) |  |
| Distal colon (D-Rs) | 52 (36.4%) | 62 (43.4%) |  |
| Rectum (Ra-Rb) | 38 (26.6%) | 37 (25.9%) |  |
| Mean period to surgery [months] | 2.4 ± 1.3 | 0.8 ± 0.9 | <0.01^‡^ |
| **Predominant histology**** |  |  | 0.91^†^ |
| Well-differentiated tubular adenocarcinoma | 130 (90.9%) | 132 (92.3%) |  |
| Moderately differentiated tubular adenocarcinoma | 12 (8.4%) | 10 (7.0%) |  |
| Poorly differentiated tubular adenocarcinoma | 0 | 0 |  |
| Mucinous | 1 (0.7%) | 1 (0.7%) |  |
| Papillary adenocarcinoma | 0 | 0 |  |
| **Lymphatic invasion**** |  |  | 1.00^†^ |
| (+) | 33 (23.1%) | 33 (23.1%) |  |
| (-) | 110 (76.9%) | 110 (76.9%) |  |
| **Venous invasion**** |  |  | 0.31^†^ |
| (+) | 33 (23.1%) | 26 (18.2%) |  |
| (-) | 110 (76.9%) | 117 (81.8%) |  |
| Other risk factors for LNM* |  |  | 0.10^†^ |
| (+) | 15 (10.5%) | 24 (17.3%) |  |
| (-)/unknown | 128 (89.5%) | 115 (82.7%) |  |
| **Depth of invasion**** |  |  | 0.20^†^ |
| pT1a | 15 (10.5%) | 9 (6.3%) |  |
| pT1b | 128 (89.5%) | 134 (93.7%) |  |
| Number of L/N evaluated (mean ± SD) | 24.7 ± 14.2 | 22.8 ± 10.7 | 0.20^†^ |
| LNM at primary/secondary surgery |  |  | 0.08^†^ |
| (+) | 7 (4.9.%) | 15 (10.5%) |  |
| (-) | 136 (95.1%) | 128 (89.5%) |  |
| Recurrence |  |  | 0.50^††^ |
| (+) | 3 (2.1%) | 6 (4.2%) |  |
| (-) | 138 (97.2%) | 137 (96.5%) |  |

Both groups showed no significant differences in the number of recurrences in propensity-score matched analysis

****Bold letter** shows the variables were used to calculate the propensity score and for the adjustments.

† Chi-square test; †† Fisher’s exact test; ‡ Student’s *t*-test; ER: endoscopic resection; SS: secondary surgery; LNM: lymph node metastasis; * Risk factors of LNM included histological findings of the poorly differentiated component, mucinous adenocarcinoma component, signet ring cell component, budding grade 2 or 3, pT1a: pathologically evaluated submucosal invasion < 1000 μm, pT1b: pathologically evaluated submucosal invasion ≥1000 μm, pStaging: pathological staging.
