# Supplementary figures and images for: Endoscopic Resection Before Surgery Does Not Affect the Recurrence Rate in Patients With High-Risk T1 Colorectal Cancer
Source: Clin Transl Gastroenterol. 2021 Apr 12;12(4):e00336. doi: 10.14309/ctg.0000000000000336 (PMC8043730; doi:10.14309/ctg.0000000000000336)

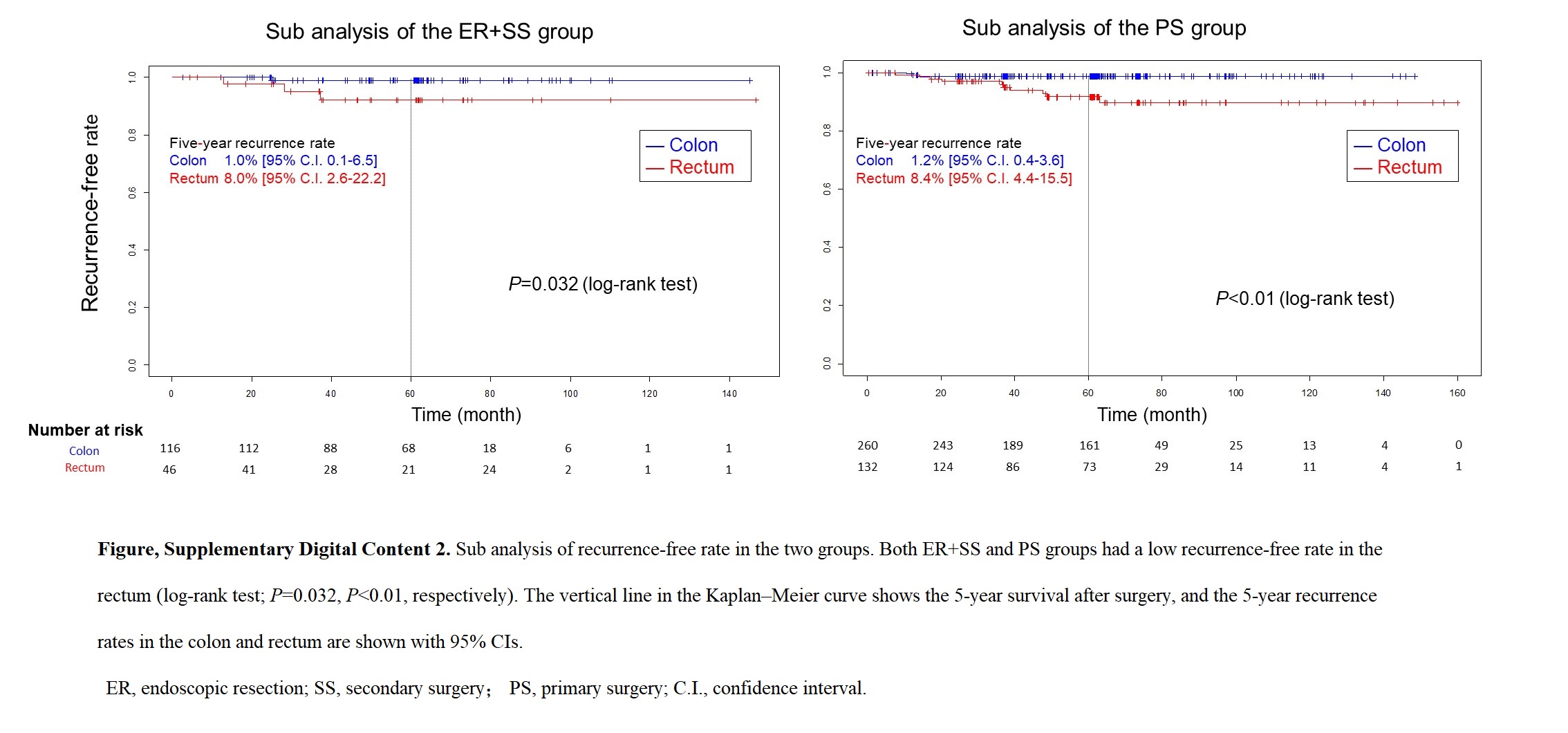

Supplement: SUPPLEMENTARY MATERIAL [file ct9-12-e00336-s003.jpg]

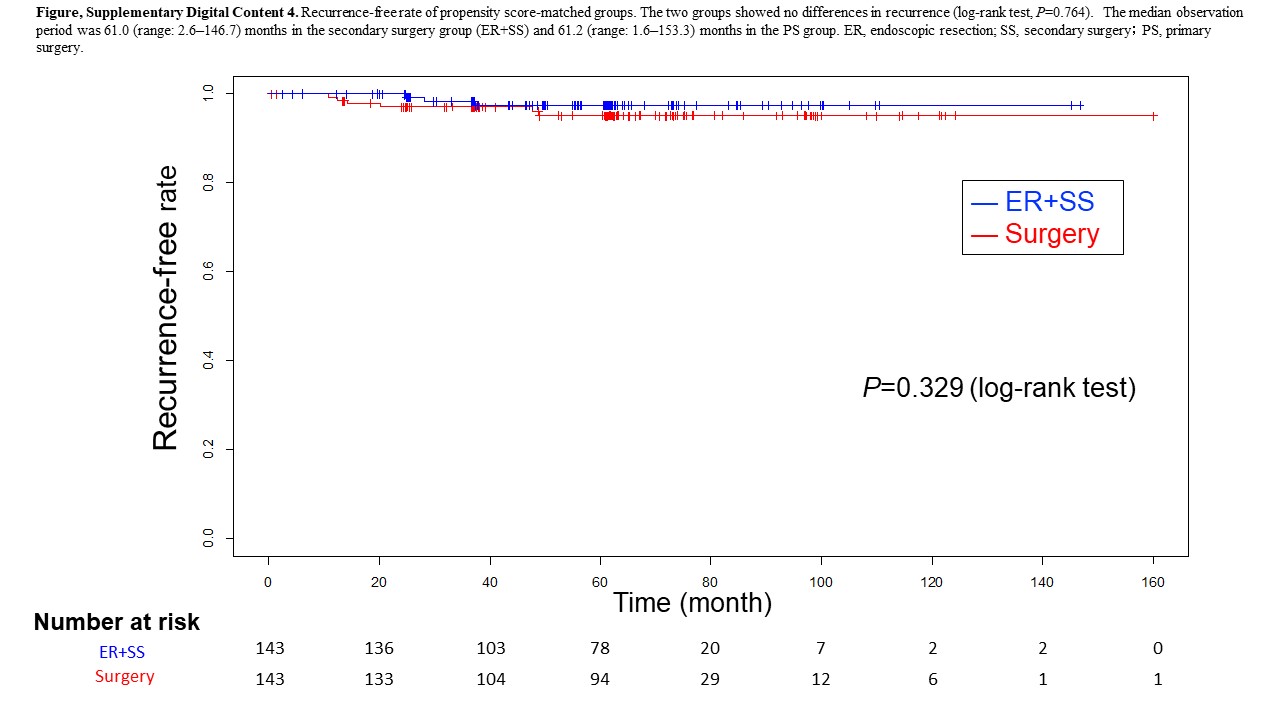

Supplement: SUPPLEMENTARY MATERIAL [file ct9-12-e00336-s004.jpg]

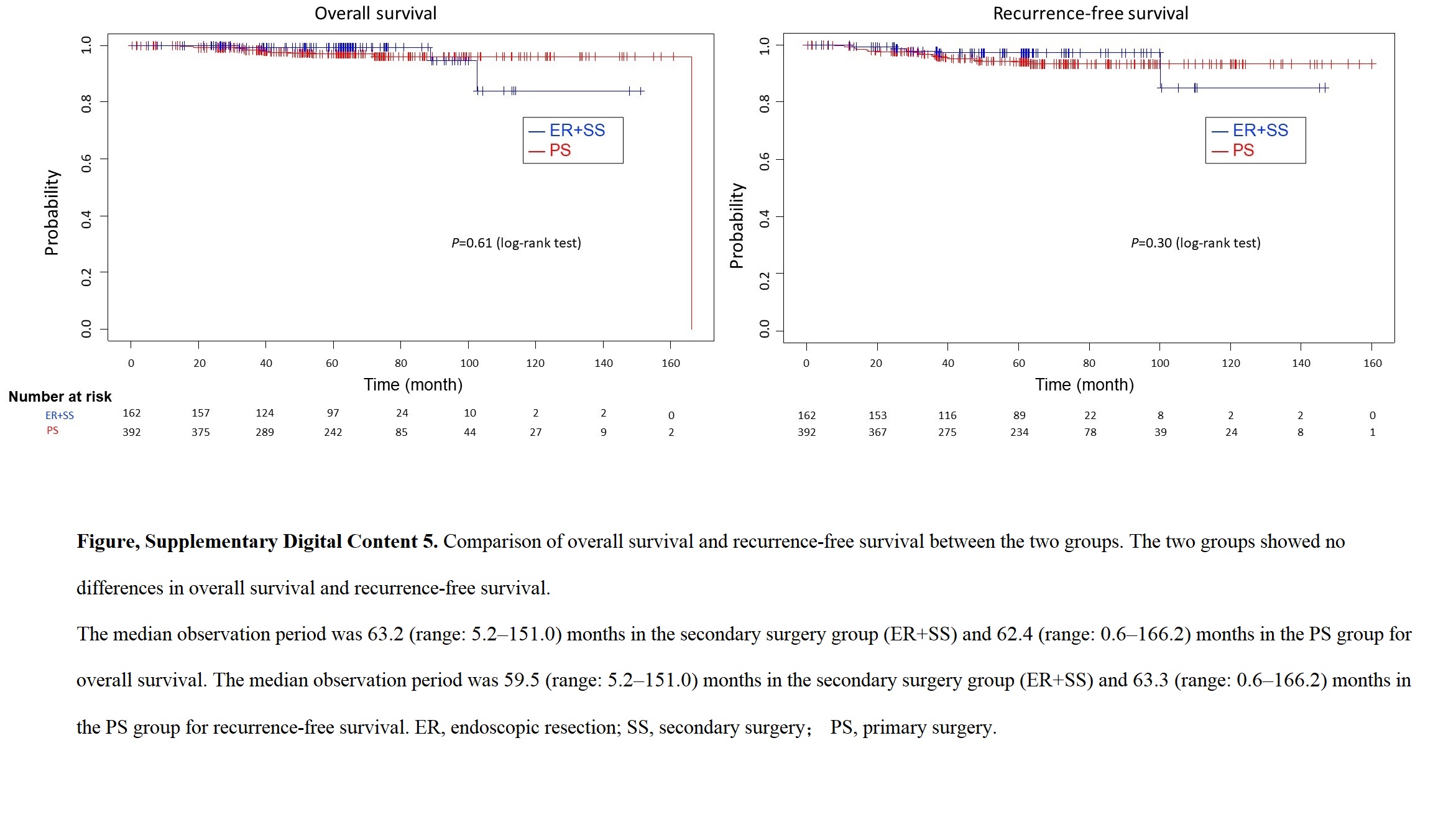

Supplement: SUPPLEMENTARY MATERIAL [file ct9-12-e00336-s005.jpg]

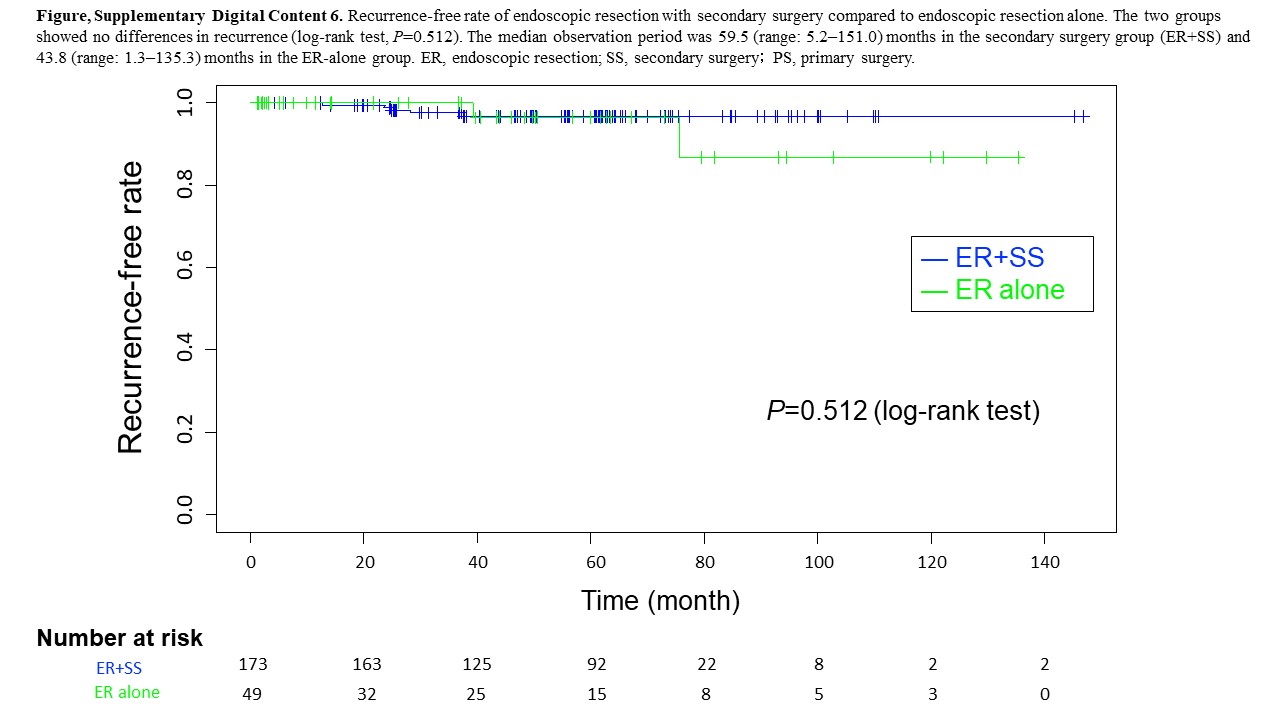

Supplement: SUPPLEMENTARY MATERIAL [file ct9-12-e00336-s006.jpg]
